# Investigating the structure of disordered eating symptoms in adult men: A network analysis

**DOI:** 10.1002/erv.3131

**Published:** 2024-08-12

**Authors:** R. Leopold Eschrich, Georg Halbeisen, Sabine Steins‐Loeber, Nina Timmesfeld, Georgios Paslakis

**Affiliations:** ^1^ University Clinic for Psychosomatic Medicine and Psychotherapy Medical Faculty Campus East‐Westphalia Ruhr‐University Bochum Luebbecke Germany; ^2^ Department of Clinical Psychology and Psychotherapy University of Bamberg Bamberg Germany; ^3^ Department of Medical Informatics Biometrics and Epidemiology Ruhr‐University Bochum Bochum Germany

**Keywords:** drive for muscularity, eating disorders, men, psychotherapy, thinness ideal

## Abstract

**Objective:**

Eating disorders (EDs) increasingly emerge as a health risk in men, but there is concern that men's symptoms go unnoticed due to stereotypical perceptions and gender‐related differences in symptom presentation. Novel assessments focused particularly on attitudes and behaviours towards increasing muscle size and definition. Using network analysis, this study aimed to corroborate and extend previous findings on disordered eating presentation in men by examining the role of muscularity concerns among an extended range of disordered eating symptoms.

**Method:**

*N* = 294 adult men (18 years or older) completed muscularity‐related and disordered eating assessments, among which we included assessments for orthorexic eating and Avoidant/Restrictive Food Intake Disorder for the first time. We selected symptoms empirically, estimated a regularised network, identified symptom communities, evaluated network loadings and bridge centrality estimates, and compared network structures between different groups of participants.

**Results:**

We identified five symptom communities related to muscularity‐related concerns, features of core ED psychopathology, and selective eating. Symptoms regarding ruminating about healthy eating, guilt for unhealthy eating, weight overvaluation, concerns about muscularity, and selective eating emerged as highly central.

**Discussion:**

The results largely corroborate previous observations but suggest that muscle‐building behaviours are part of a broader cluster of male body shaping and rule‐based dieting behaviours.

## Key points

- This network analysis aimed to corroborate and extend previous findings on disordered eating presentation in men by examining the role of muscularity concerns among an extended range of disordered eating symptoms.
- We identified five symptom communities related to muscularity‐related concerns, features of core Eating disorder (ED) psychopathology, and selective eating.
- The most central symptoms were ruminating about healthy eating, guilt for unhealthy eating, weight overvaluation, concerns about muscularity, and selective eating.

## 1 INTRODUCTION

Eating disorders are characterised by body image concerns, disturbances in eating, and weight‐control behaviours (American Psychiatric Association, 2013) and have been placed among the most severe mental disorders in terms of psychological burden and mortality (Treasure et al., 2020). Contrasting stereotypical perceptions, EDs increasingly emerge as a health risk in men (Nagata et al., 2021). Prevalence and burden have risen faster in men than women over the last 30 years (Ferrari, 2022). Globally, up to 8.4% of women and 2.2% of men develop EDs such as Anorexia Nervosa, Bulimia Nervosa, or Binge‐eating Disorder during their lifetime (Galmiche et al., 2019; Santomauro et al., 2021). Thus, men may constitute up to every fourth clinical ED case.

Men, however, remain underrepresented in ED research and care (Halbeisen, Brandt, & Paslakis, 2022). There is concern that persistent stigma against men with EDs delays help‐seeking behaviours (Richardson & Paslakis, 2021), but also that men's symptoms go unnoticed by healthcare professionals due to gender differences in symptom presentation (Matsumoto & Rodgers, 2020). Most standard psychopathology assessments were developed based on ED presentation in girls and women and usually do not assess symptoms potentially more relevant for men (Gallagher et al., 2021; Murray et al., 2017). For example, some men are less concerned with thinness and seek to increase body mass and muscularity (Lavender et al., 2017). These differences in presentation raised validity concerns (e.g., Laskowski et al., 2023) and could also account for men's lower symptom endorsement in standard ED assessments (Halbeisen, Braks, et al., 2022; Rica et al., 2021; Schaefer et al., 2018). Therefore, we must advance our understanding of diverse ED presentations and develop appropriate screening and assessment tools for men (Byrne et al., 2023).

### 1.1 The role of muscularity concerns

Recently developed ED assessments in men focus mainly on attitudes and behaviours towards increasing muscle size and definition (Cooper et al., 2020; Díaz de León Vázquez et al., 2022; Forbush et al., 2013; Murray et al., 2019; Stanford & Lemberg, 2012). The central role of muscularity for men's disordered eating received support from network analysis, an increasingly popular approach for analysing symptom interactions (Borsboom et al., 2021; Monteleone & Cascino, 2021). Network analysis represent specific symptoms as *nodes* (e.g., body dissatisfaction, driven exercise) and symptom interactions as *edges* (connections) between nodes (e.g., pairwise partial correlations). The network's arrangement, its topology, can be evaluated to detect communities of highly connected symptoms, akin to factors in factor analyses, and to quantify how strongly individual nodes influence others, which is a way of assessing a symptom's centrality. Network analyses thus provide insights into the structure of disordered eating symptoms in men and the importance of muscularity concerns.

Forrest et al. (2019) conducted a network analysis with ED psychopathology and muscularity concerns assessed in men classified as having a low or high risk for an ED. Guilt associated with missed training sessions and the use of supplements emerged as the most central (i.e., influential) symptoms across groups, together with weight/shape overvaluation and the fear of losing control over eating. Similarly, Prnjak et al. (2021), using community detection algorithms, found a symptom network of body image disturbances (weight/shape overvaluation), leanness concerns (i.e., having low body fat), and two separate muscularity‐related attitudes and behaviours communities in men and women. Central symptoms connecting communities (called *bridge nodes*) were positive attitudes towards gaining muscles and weightlifting behaviour, and “traditional” symptoms related to body image disturbances (hating one's body) and leanness (striving for toned muscles). Together, the findings suggest that muscularity concerns constitute a uniquely identifiable and crucial diagnostic target in men, possibly contributing to ED development and maintenance (we must note that previous network analyses that featured women mainly or exclusively, rarely included muscularity concerns; thus, muscularity concerns could be similarly significant in women; Tomei et al., 2022).

However, the extent to which network results are reproducible requires further investigation. Network results highly depend on the included variables (Monteleone & Cascino, 2021). Detected communities and centrality estimates can change when including hitherto unaccounted symptoms (e.g., by removing spurious correlations; Prnjak et al., 2021). Thus far, network analyses with men have included mostly thinness‐ or muscularity‐related measures. While arguably important (Schaefer et al., 2021), other concerns, such as the obsessive preoccupation with healthy eating (Orthorexia Nervosa, ON), could independently account for both restrictive eating (Atchison & Zickgraf, 2022) and muscle‐building behaviours (MacPhail & Oberle, 2022; Merhy et al., 2023), and should thus be included in men's symptom networks. Some men (and women) may also restrict eating based on sensory sensitivities or eating‐related anxieties, which has been described as Avoidant/Restrictive Food Intake Disorder (ARFID; American Psychiatric Association, 2013). To the best of our knowledge, no study has yet explored muscularity concerns in the presence of ARFID in symptom networks, and their inclusion in addition to “traditional” disordered eating symptoms could thus further illuminate the structure of disordered eating symptoms in men.

### 1.2 The present study

This study aimed to corroborate and extend previous findings on disordered eating presentation in men by examining the role of muscularity concerns among an extended range of ED symptoms using network analysis. Specifically, we wanted to examine how muscularity concerns and which specific symptoms link to disturbed eating patterns, body image disturbances, and weight‐control behaviours, among which we included ON and ARFID symptoms for the first time. While we expected overall positive associations between the included disordered eating symptoms and muscularity concerns, we had no a priori predictions concerning the centrality of individual symptoms in the network structure. To inform the development of screening and assessment tools, we also compared ED symptoms and networks between men with low and high risk for having an ED (Forrest et al., 2019). While we expected men with an increased ED risk to show pronounced ED symptoms, muscularity concerns, and ON symptoms, previous findings gave no clear indication of how other symptoms or network structures could differ. Our investigation thus partly remained exploratory.

## 2 METHODS

### 2.1 Participants

We recruited two samples of adult men (≥18 years) for a cross‐sectional online survey. A first convenience sample was recruited among university and social network forums and among acquaintances between May 2022 and June 2023. A second sample was recruited using Prolific (www.prolific.com) on 8 April 2024. We initially targeted to collect data for one year or up to 200 participants but then increased our target to *N* = 300 based on simulation‐based recommendations that “sample sizes ranging from 250 to 350 are generally enough to observe moderate sensitivity, high specificity, and high edge weights correlation when the networks are sparse and consist of 20 nodes or less” (Constantin & Cramer, 2019). Power decreases with a higher number of nodes and non‐zero edges (Forrest et al., 2019), but increases when moving from “random” towards more systematically structured networks (Prnjak et al., 2021). We, therefore, settled for following the general recommendation given that we could not specify a more specific network structure a priori.

The study received ethics approval (AZ 2022‐910, 21 April 2022), was registered at https://aspredicted.org/5T3_NH5, and was carried out in accordance with the Declaration of Helsinki. All participants gave informed consent. Participants of the convenience sample did not receive compensation. Participants recruited via Prolific received £4.50 for their participation. We report all measures and exclusions. Data and materials can be obtained from the corresponding author upon request.

### 2.2 Measures

#### 2.2.1 Sociodemographic variables

Participant self‐reported their age, weight, height, regular medications, sex (male, female, “diverse” [i.e., intersex, the legally‐recognized third gender in Germany]), sexual orientation (towards men, women, men and women, other genders not listed, no answer), German ability (native, fluent, basic), migration background (yes, no), years of education (<12 years, >12 years), marital status (single, married, divorced, widowed), living circumstances (alone, with others), history of EDs (open‐ended), ongoing ED treatment (open‐ended), and previous study participation (yes vs. no). The latter question served to exclude datasets from repeated participation.

#### 2.2.2 Eating disorder risk

To stratify the sample based on ED risk, participants completed the German short version of the Eating Attitudes Test (EAT‐8; Richter et al., 2016), one of the few available screening instruments with validated cut‐off values for men. The EAT‐8 includes eight yes‐or‐no questions regarding core ED symptoms, with “yes” sum scores ≥2 serving as a conservative cut‐off value (Kuder‐Richardson's KR‐20 = 0.74).

#### 2.2.3 Symptom assessment

##### Disordered eating

As in previous studies (Forrest et al., 2019; Prnjak et al., 2021), we used the validated German version of the Eating Disorder Examination‐Questionnaire (EDE‐Q; Fairburn & Beglin, 1994; Hilbert et al., 2007). The EDE‐Q assessed cognitive and behavioural ED symptoms within the last 28 days along four subscales, “(Dietary) Restraint”, “Eating Concern”, “Shape Concern”, and “Weight Concern”, using 22 attitudinal items rated on a 7‐point scale (from 0, never, to 6, every day). Six additional items assessed overeating episodes, binge episodes, binge days, and purging behaviours, that is, vomiting, laxative use, and driven exercise. Since previous investigations did not support the proposed factor structure of the EDE‐Q in men (Laskowski et al., 2023), we did not consider the subscale scores and only reported the global mean score across attitudinal items (Cronbach's *α* = 0.90).

The Duesseldorf Orthorexia Scale (DOS; Barthels et al., 2015) captured ON, the fixation on healthy nutrition and associated behaviours, over the previous 7 days with 10 statements (e.g., I prioritise healthy eating over pleasure) rated on a 4‐point scale (from 1, does not apply at all, to 4, fully applied). We report the scale mean across all items (Cronbach's *α* = 0.85).

The Eating Disorders in Youth‐Questionnaire (EDY‐Q; van Dyck & Hilbert, 2016), adapted for adults and the only available German ARFID questionnaire (Hilbert et al., 2021), assessed ARFID symptoms using 14 questions covering food avoidance (FA), selective eating (SE), functional dysphagia (FD), and problems with underweight. Additional questions assessing pica, rumination, and weight/shape concerns (ARFID exclusion criteria) are not used for scale construction. All items were rated on a 7‐point scale (from 0, never, to 6, always). We report the EDY‐Q subscale and total mean scores (Cronbach's *α* = 0.69).

##### Muscularity and leanness concerns

The validated German version of the Muscle Dysmorphic Disorder Inventory (MDDI; Zeeck et al., 2018) included 13 items on muscularity‐related body image disturbances and behaviours rated on a 5‐point scale (from 1, never, to 5, always), that is, the drive for size (DS, e.g., body too skinny; *α* = 0.88), appearance intolerance (AI, e.g., hate my body; *α* = 0.87), and functional impairment (FI, e.g., depressed if not exercising; *α* = 0.86). We report the subscale and MDDI total means (Cronbach's *α* = 0.83).

The Visual Body Scale for Men (VBSM; Talbot et al., 2019) is a figural rating scale designed to measure participants' body dissatisfaction regarding muscularity (VBSM‐M) and leanness, that is, body fat (VBSM‐BF). The VBSM‐M consisted of 10 male bodies differing in increasing muscularity, and the VBSM‐BF of 10 male bodies depicting a continuous increase in BF percentage. For each scale, participants first indicated which body best corresponds to their actual body, followed by selecting the body that best corresponds to their ideal body, with actual‐ideal discrepancy scores serving as the primary outcomes.

#### 2.2.4 Additional questions

Further exploratory questions, not reported here, assessed therapy motivation and stereotypical perceptions of men with EDs (Lehe et al., *manuscript under review*).

### 2.3 Procedure

We implemented the online study on our web server using jsPsych (de Leeuw et al., 2023). Upon assessing the study's website, participants read the study information and consent forms. We explained that the study concerned EDs in men and that participants could receive feedback on their ED risk. After providing informed consent, participants completed the sociodemographic questionnaire and the ED risk assessment. The remaining questionnaires were completed next, in randomized order. If participants consented to receive feedback, the EAT‐8 score and an explanatory text were displayed. If participants did not consent to receive feedback, they directly advanced to the last screen and were thanked and dismissed. We estimated the study to be completed in 15–20 min.

### 2.4 Data analysis

Closely following and building upon previous studies (Forrest et al., 2019; Prnjak et al., 2021), we initially characterised and compared ED and associated symptom profiles between men with low and high ED risk, using the validated scales' overall and subscale scores. We used independent samples *t*‐tests when comparing continuous variables, Pearson correlations for associations, and Chi‐squared (χ^2^) frequency tests when comparing categorical variables, with a significance threshold of *p* < 0.05, in SPSS 28 (IBM Corp, 2021). We screened for multivariate outliers across questionnaire scores using Mahalanobis distance with a criterion of *p* < 0.001 to detect irregularities in the data (for example, from people just clicking through the questionnaires) and examined minimum completion times before analysis.

We then estimated participants' symptom networks using R 4.3.3 (R Core Team, 2022). Specifically, we estimated Gaussian Graphical Models based on Pearson correlations via the *qgraph* package 1.9.8 (Epskamp et al., 2012) with glasso (graphical least absolute shrinkage and selection operator) regularisation and EBIC (Extended Bayesian Information criterion) tuning parameter selection (*γ* = 0.5), which reduces false discovery rates compared to the classical BIC (Foygel & Drton, 2010). This yields a sparse, undirected network of conditional associations between included symptoms (i.e., controlled for all other symptoms in the network), with small and unstable edges set to zero (Epskamp & Fried, 2018). Before estimating the network, all items were standardized based on their scale range, and missing responses (0.9% overall) were imputed using predictive mean matching via the *mice* package 3.16.0 (van Buuren & Groothuis‐Oudshoorn, 2011).

The network estimation involved the following steps: First, we selected items to serve as network nodes from all assessments with the ‘goldbricker’ and ‘net_reduce’ functions in *networktools* 1.5.2 (Jones, 2023). The functions identify and remove redundant item pairs (those with less than a specified threshold proportion of significantly different correlations with the remaining items), given that multicollinearity may obscure the true symptom structure. We used the default significance and proportion threshold parameters (0.05 and 0.25, respectively), and kept the first principal components of the redundant pairs as new variables (i.e., the ‘principal component analysis’ method, preserving information about which items were redundant in the final results).

Second, we determined symptom communities via ‘bootEGA’ in *EGAnet* 2.0.5 (Golino & Christensen, 2023; Golino & Epskamp, 2017). The function derives the typical network structure from the median values of edges across 1000, non‐parametric bootstrapped samples, and then uses ‘Walktrap’ to detect symptom communities. Using bootstrapped community detection represented a crucial improvement, as it empirically assessed the stability of communities and items (how often one detects a specific number of communities and replicates the items assignment across bootstrap iterations). Simulations show that items with replication rates lower than 0.75 can be considered ‘unstable’ (Christensen & Golino, 2021b), which we therefore removed before proceeding.

Third, we determined centrality estimates for the average (bootstrapped) network of the final item selection. Going beyond previous studies, we computed two types of symptom centrality estimates within and between communities: network loadings and bridge centrality. Network loading, extracted from the average network using ‘net.loads’ in *EGAnet*, index the sum of connections of a node to other nodes of the same community; bridge strength and bridge expected influence (EI), the recommended bridge centrality indices extracted via ‘bridge’ in *networktools*, instead index connections between nodes of different communities (Jones et al., 2021). Bridge strength is the sum of absolute edge values, which reflects the extent to which one node influences nodes from other communities. Bridge EI is the sum of positive and negative edges, which thus reflects the overall direction of influence of one node onto nodes of other communities (we did not compute node strength or node EI since these measures do not take the clustering of symptoms into distinct communities into account and thus potentially conflate two conceptually distinct sources of covariation). Because networks are estimated with some degree of error, we also determined the stability of centrality estimates via *bootnet* 1.6 (Epskamp et al., 2018) using *CS*‐coefficients, which quantify the proportion of cases that can be dropped to retain, with 95% certainty, a correlation with the original centrality parameters higher than 0.70. To interpret centrality parameters, *CS*‐coefficients should not be below 0.25, and preferably above 0.5. We also used the *bootnet* package's non‐parametric, bootstrapped difference test to evaluate differences in centrality parameters.

Finally, and similar to Prnjak et al. (2021), we used *NetworkComparisonTest* 2.2.2 (van Borkulo et al., 2022) to compare symptom networks between men with high and low ED risk. This permutation‐based hypothesis test used the selected nodes and communities from the overall network (see above) and assessed the difference between estimated networks in the high‐ and low‐risk groups. The test provides several invariance measures, evaluated against a random sampling of two groups from the overall (high and low risk) datasets, of which we examined network structure invariance (which evaluates if all edges are equal) and global strength invariance (which evaluates if the overall level of connectivity is the same across risk groups).

## 3 RESULTS

### 3.1 Sample characteristics

We recruited 308 participants in total; 294 men (*M*_age_ = 33.11, age range: 18–73 years) fulfiled all inclusion criteria (no multivariate outliers detected; completion times ranged from 6 to 74 min, *M* = 18.45, SD = 10.35). The convenience and Prolific samples did not differ regarding age, BMI, or sociodemographic features, except that the Prolific sample was more diverse regarding migration background, and, consequently, fewer participants were German native speakers (see Table 1). The EAT‐8 classified *n* = 68 as low risk for having an ED and *n* = 226 as high risk (the rates did not differ between the convenience and Prolific sample, *p* = 0.09). High‐risk participants had a higher BMI than low‐risk participants, *t*(292) = 4.13, *p* < 0.001, *d* = 0.57. However, they were of similar age, *p* = 0.83. Eating disorder risk was not associated with other demographic categories, all *p*s > 0.05.

**TABLE 1:** Sample characteristics.

Parameter	Total	Convenience	Prolific
*n*	294	146	148
Age (years)	33.1 (11.6)	33.8 (12.8)	32.4 (10.3)
BMI (kg/m^2^)	26.48 (6.3)	26.5 (6.8)	26.46 (5.9)
Gender			
Male	292	145	147
Diverse	2	1	1
Sexual preference			
Women	249	123	126
Men	28	15	13
Women and men	13	6	7
Other	1	1	0
No comment	3	1	2
German language			
First language	265	142	123
Fluent	26	4	22
Basic	3	0	3
Education			
Less than 12 years	45	27	18
12 years or more	249	119	130
Migration background			
Yes	69	17	52
No	225	129	96
Marital status			
Married	78	40	38
Single	208	100	108
Divorced	8	6	2
Living situation			
Alone	82	44	38
With others	212	102	110
Current ED treatment			
Yes	23	14	9
No	179	83	96
No response	92	49	43

*Note*: High and low risk groups based on EAT‐8 sum scores (≥2 for high risk, <1 for low risk).

### 3.2 Symptom profiles

Table 2 displays the total and risk groups' means, and comparison statistics. Consistent with the screening categorisation, the high‐risk group presented with higher EDE‐Q, DOS, and MDDI global scores than the low‐risk group. However, of the MDDI subscales, only FI and AI were elevated, but not DS. Similarly, men with high risk showed pronounced leanness discrepancies (VBSM‐BF) but not muscularity discrepancies (VBSM‐M; although men with high risk endorsed more muscular ideals than men with low risk, they also rated themselves as more muscular). Eating Disorders in Youth‐Questionnaire food avoidance was elevated in the high‐risk group; EDY‐Q total and subscale scores did not differ.

**TABLE 2:** Questionnaire means (standard deviations in parentheses) and comparisons.

Variable	Total (*N* = 294)	High risk (*n* = 226)	Low risk (*n* = 68)	*t*	*p*	*d*
EDE‐Q	1.55 (1.26)	1.85 (1.26)	0.55 (0.55)	8.24	<0.001	1.14
DOS	0.84 (0.54)	0.96 (0.53)	0.44 (0.30)	7.62	<0.001	1.05
EDY‐Q*	1.32 (0.83)	1.32 (0.83)	1.32 (0.81)	−0.01	1.00	0.00
FA	1.43 (1.14)	1.51 (1.15)	1.17 (1.06)	2.09	0.04	0.30
SE	1.96 (1.46)	1.96 (1.45)	1.95 (1.50)	0.01	0.99	0.00
FD	0.27 (0.81)	0.31 (0.86)	0.14 (0.60)	1.46	0.15	0.21
MDDI	1.19 (0.66)	1.30 (0.65)	0.85 (0.58)	5.14	<0.001	0.71
DS	1.36 (0.95)	1.36 (0.92)	1.36 (1.05)	−0.02	0.98	0.00
AI	1.37 (1.07)	1.58 (1.08)	0.68 (0.63)	6.49	<0.001	0.90
FI	0.80 (0.84)	0.93 (0.88)	0.37 (0.52)	5.05	<0.001	0.70
VBSM‐BF	1.20 (2.38)	1.68 (2.27)	−0.38 (2.04)	6.71	<0.001	0.93
VBSM‐M	1.79 (2.18)	1.74 (2.28)	1.96 (1.81)	−0.72	0.47	−0.10

*Note*: *N = 278 (high risk: *n* = 213; low risk: *n* = 65) due to missing responses. High and low risk groups based on EAT‐8 sum scores (≥2 for high risk, <1 for low risk).

Abbreviations: AI, appearance intolerance; BF, body fat; DOS, Duesseldorf Orthorexia Scale; DS, drive for size; EDE‐Q, Eating Disorder Examination‐Questionnaire; EDY‐Q, Eating Disorders in Youth‐Questionnaire; FA, food avoidance; FD, functional dysphagia; FI, functional impairment; M, muscularity; MDDI, Muscle Dysmorphic Disorder Inventory; SE, selective eating; VBSM, Visual Body Scale for Men.

### 3.3 Network estimation

All scale‐building items of the EDE‐Q (plus binge episodes and driven exercise), DOS, MDDI, EDY‐Q, and the VBSM discrepancy scores were evaluated for multicollinearity (we excluded EDE‐Q´s laxative abuse and self‐induced vomiting as fewer than 5% of participants reported these behaviours; we also coded binge episodes as missing prior to imputation that exceeded the reported number of overeating episodes, which indicated incorrect answering in the EDE‐Q). Goldbricker recombined 13 redundant pairs of the original 59 items, leaving 46 (combined) items. bootEGA then identified another 12 items with replication indices lower than 0.75, showing they cannot be consistently assigned to a specific symptom community. We removed these items, leaving a final selection of 34 for the remaining analyses (13 EDE‐Q [5 combined], including binge episodes and driven exercising; 5 DOS [1 combined]; 9 MDDI [3 combined]; 5 EDQ‐Y; and VBSM‐M and VBSM‐BF).

**FIGURE 1:**
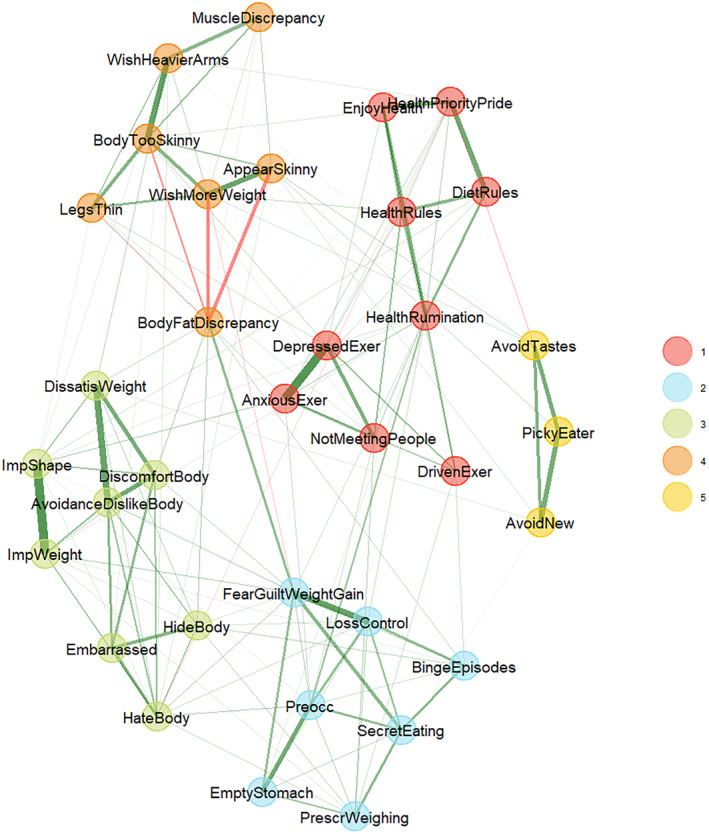
The average disordered eating symptom network in adult men. Thicker lines represent stronger edges (regularised conditional correlations), with green denoting positive and red negative edges. Node colours signify empirically‐determined symptom communities.

Figure 1 shows the median network. The items consistently formed five symptom communities in 95% of cases across 1000 bootstrap samples, with all item replication indices ≥0.96. Community 1 included items of the DOS related to obsessive healthy eating, the EDE‐Q's driven exercise item, and MDDI items related to FI due to missed training. Community 2 included EDE‐Q items related to fear of weight gain, avoidance of social eating and eating in secret, and binge episodes. Community 3 included EDE‐Q and MDDI items that addressed body dissatisfaction and shape and weight overvaluation. Community 4 captured the MDDI's “drive for size”, muscularity‐related body image discrepancies (VBSM‐M), EDY‐Q underweight concerns, and—associated *negatively* with the other symptoms—body fat‐related body image discrepancies (VBSM‐BF). Finally, Community 5 included the EDY‐Qs selective eating items.

**FIGURE 2:**
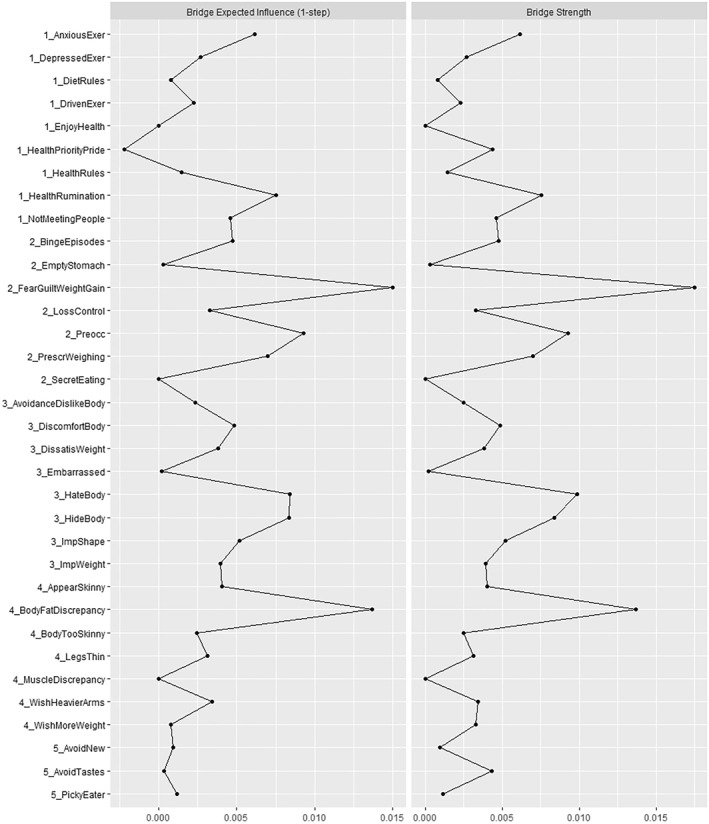
Normalised bridge centralities values ordered by symptom community. Bridge strength is the sum of absolute edge values, which reflects the extent to which one node influences nodes from other communities; bridge expected influence (EI) is the sum of positive and negative edges, which thus reflects the overall direction of influence of one node onto nodes of other communities.

Table 3 shows the network loadings (within‐community centrality), and Figure 2 the bridge (between‐community) centrality estimates. The most central (i.e., highest‐loading) symptoms within communities 1–5 were feeling depressed when missing a training session, eating in secret, discomfort seeing one's body, feeling too skinny, and being a picky eater, respectively. The most important symptoms in terms of absolute influence on other communities (bridge strength) were ruminating about healthy eating (Community 1), feeling guilty for unhealthy eating and fearing weight gain (Community 2), hating one's body (Community 3), experiencing BF‐related discrepancies (Community 4), and being avoidant of specific tastes or other features (Community 5). Comparison tests showed that bridge strength estimates for these nodes did not differ significantly, except that feeling guilty for unhealthy eating/fearing weight gain had significantly more strength than ruminating about healthy eating and avoiding tastes (see Supporting Information Figure S1). Bridge EI yielded almost identical results, except that in Community 5, being picky emerged as a slightly more positive influencer of other symptoms than avoiding specific tastes. Notably, Community 1 contained the only node with a descriptively negative EI (prioritising and taking pride in healthy eating), suggesting this symptom “deactivates” symptoms from other communities. Again, the bridge EI values of the most central nodes were not statistically different, except that feeling guilty for unhealthy eating/fearing weight gain and BF‐related discrepancies outperformed the influence of picky eating. *CS*‐coefficients were consistently larger than 0.50, suggesting that the centrality estimates were interpretable (0.71 for edge strength, 0.67 for bridge EI and bridge strength, respectively; because CS‐coefficients for network loadings are not available, we report edge strength, which combines network and bridge strength loadings; Christensen & Golino, 2021a).

**TABLE 3:** Network loadings of items across communities.

Variables	Scale IDs	Description	Com1	Com2	Com3	Com4	Com5
DepressedExer	MDDI 12	Feeling depressed when missing exercise	0.35				
AnxiousExer	MDDI 10	Feeling anxious when missing exercise	0.32				
HealthRumination	DOS 8	Thoughts revolve around healthy nutrition	0.30				
HealthRules	DOS 9	Difficulty going against dietary rules	0.28				
HealthPriorityPride	DOS 5 + DOS 1	Liking to pay more attention to healthy nutrition + health more important than enjoyment	0.27				
NotMeetingPeople	MDDI 13 + MDDI 11	Missing opportunities to meet new people + cancelling social activities for workout	0.26				
EnjoyHealth	DOS 3	Enjoying only healthy foods	0.26				
DietRules	DOS 2	Adherence to nutritional rules	0.25				
DrivenExer	EDE‐Q 18	Frequency of driven exercising	0.16				
SecretEating	EDE‐Q 21 + EDE‐Q 19	Social eating + eating in secret		0.36			
LossControl	EDE‐Q 9	Fear of losing control over eating		0.35			
FearGuiltWeightGain	EDE‐Q 20 + EDE‐Q 10	Guilt about eating + fear of weight gain		0.34			
Preocc	EDE‐Q 8 + EDE‐Q 7	Preoccupation with shape or weight + preoccupation with food, eating, or calories		0.30			
EmptyStomach	EDE‐Q 5	Empty stomach		0.25			
BingeEpisodes	EDE‐Q 14	Binge episodes		0.17			
PrescrWeighing	EDE‐Q 24	Reaction to prescribed weighting		0.16			
AvoidanceDislikeBody	EDE‐Q 28 + EDE‐Q 25	Avoidance of exposure + dissatisfaction with weight			0.42		
DiscomfortBody	EDE‐Q 27	Discomfort seeing body			0.35		
ImpWeight	EDE‐Q 22	Importance of weight			0.30		
DissatisWeight	EDE‐Q 26	Dissatisfaction with shape			0.30		
Embarrassed	MDDI 9	Embarrassed without shirt			0.27		
ImpShape	EDE‐Q 23	Importance of shape			0.27		
HateBody	MDDI 3	Hating own body			0.26		
HideBody	MDDI 2	Wearing loose clothing			0.18		
BodyTooSkinny	MDDI 5 + MDDI 1	Finding chest too small + thinking body is too slender				0.45	
WishMoreWeight	EDY‐Q 5	Wishing to have more weight				0.40	
WishHeavierArms	MDDI 8 + MDDI 4	Wishing arms stronger + wishing to be heavier				0.29	
AppearSkinny	EDY‐Q 4	Others think too skinny				0.28	
LegsThin	MDDI 6	Feeling legs too thin				0.23	
MuscleDiscrepancy	VBSM‐M	Muscularity perceived/desired discrepancy				0.15	
BodyFatDiscrepancy	VBSM‐BF	Body fat perceived/desired discrepancy				−0.25	
PickyEater	EDY‐Q 8	Being picky					0.40
AvoidNew	EDY‐Q 9	Avoid trying new foods					0.37
AvoidTastes	EDY‐Q 12	Avoid foods for taste, consistency, looks, or smell					0.36

*Note*: Network loadings represent each node's (unique) contribution to the emergence of a symptom community.

Abbreviation: EDY‐Q, Eating Disorders in Youth‐Questionnaire.

Finally, we compared symptom networks between men with high and low ED risk using the nodes and communities from the overall network. Between risk groups, the network structure invariance test was not significant, *M* = 0.51, *p* = 0.68. Similarly, the global strength invariance test was not significant, *S* = 14.89, *p* = 0.81. Thus, there were no indications that edges or the level of connectivity differed between high‐ and low‐risk groups. However, although the centrality estimates in the high‐risk network were sufficiently stable (*CS*‐coefficients: 0.58 for edge strength, 0.52 for bridge EI and bridge strength, respectively), we could not estimate the stability of the low‐risk network given the limited sample size. Thus, comparisons should be interpreted with caution.

## 4 DISCUSSION

Previous network analyses support the assumed central role of muscularity concerns in men's disordered eating symptom networks, but uncertainty remained regarding their reproducibility when including hitherto unaccounted symptoms. We, therefore, aimed to corroborate and extend previous findings by examining the role of muscularity concerns among an extended range of ED symptoms, including ON (via the DOS) and ARFID (via the EDY‐Q) for the first time.

Consistent with our hypothesis of overall positive associations between the included disordered eating symptoms and muscularity concerns, the empirically selected items from validated measurements formed five symptom communities that included, among others, muscularity concerns and aspects of core ED psychopathology (Fairburn et al., 2003) that largely corroborated previous observations. Similar to Prnjak et al. (2021), Community 1 included muscularity‐related behaviours (e.g., feeling depressed when missing a training), and Community 4 comprised muscularity‐related attitudes (e.g., feeling too skinny) in the absence of BF‐related body image discrepancies. These were complemented by selective eating tendencies (Community 5, i.e., the EDY‐Qs selective eating items) and what Forrest et al. (2019) described as ED‐specific forms of fear and avoidance (Community 2, e.g., eating in secret and avoiding social eating) and shape/weight overvaluation (Community 3, e.g., discomfort seeing one's body). However, our results suggest further refining the cluster's characterisation, especially regarding muscularity‐related behaviours. Rather than exclusively addressing muscle‐building (as in Prnjak et al., 2021) or “just” muscle dysmorphia co‐morbid with disordered eating (Badenes‐Ribera et al., 2019), the extension of symptoms showed that Community 1 combined exercising and healthy eating, that is, different body shaping and rule‐based dieting behaviours. Notably, the community included none of the dieting and weight‐control behaviours typically associated with EDs (e.g., the EDE‐Q´s eating restraint, eating avoidance, and FA), leading us to speculate that Community 1 could reflect phenotypically “male” body shaping and rule‐based dieting beyond muscularity‐related manifestations (i.e., including, but not limited to eating for muscle building; Lavender et al., 2017), separate from “female” dieting and weight‐control behaviours such as counting calories or restricting food intake. Similar selective eating tendencies were still present among men's symptom network, but relegated to Community 5, see above, and thus occurred largely independent from both muscularity and thinness concerns. Interestingly, the presence of ARFID‐associated selective eating tendencies further demonstrates that “picky eating” is not limited to early childhood and may emerge later in life (Lange et al., 2019). Considering ARFID symptoms could thus help to identify disordered eating in men.

Similar patterns also emerged regarding symptom centrality. Feeling depressed for missed training sessions emerged as a highly central symptom in Community 1 (Forrest et al., 2019), and ruminating about healthy eating from the DOS (which had not been included in previous studies) emerged as the strongest bridge. Feeling guilty for unhealthy eating combined with fear of weight gain was also highly central within Community 2 (Forrest et al., 2019), and emerged as a strong bridge to symptoms from other communities. These patterns corroborate the observed contribution of emotion regulation difficulties to ED psychopathology networks (Monteleone & Cascino, 2021) and thus highlight that psychopathological features non‐specific to EDs should be further explored (Krehbiel et al., 2021). For example, repressing and internalising negative affect can aggravate body dissatisfaction (Momeñe et al., 2023), which could explain the observed symptom association. This interpretation in terms of dysregulation is further consistent with the observation that *positive* emotions associated with Community 1 (taking pride in healthy eating) were linked to reduced activation in other symptom communities.

Other centrality estimates were also consistent with previous investigations. Avoiding and disliking one's body best characterised Community 3, and hating one's body emerged as the strongest bridge (Prnjak et al., 2021). This is also consistent with networks of mixed‐gender samples (Wang et al., 2019) and with Forrest et al., who observed a prominent role of shape overevaluation in influencing other symptoms (Forrest et al., 2019). In a similar vein, the relatively high centrality of feeling too skinny within Community 4 mirrored the central role of positive attitudes towards “bulking up” found in Prnjak et al. (2021), but more specific attitudes towards arms and legs (rather than the more general attitude) had a greater influence on other symptom communities. This finding may appear somewhat arbitrary until acknowledging the anecdotal evidence that, for example, leg and calf muscles are often characterised as the most difficult to grow within fitness communities (indeed, the most prominent indication for calf augmentation is aesthetic concerns; Escandón et al., 2022). Thus, one could speculate that obsessing about these exposed muscles indicates an already advanced and, therefore, a particularly influential form of muscularity‐related attitudes. However, we must note that we did not measure or control for “objective” body mass or musculature, which may limit conclusions in terms of body image distortion.

Moreover, perceived BF discrepancies, which correlated negatively with other muscularity‐related attitudes, exerted the strongest influence on other symptom communities. This could be interpreted as a counter‐regulation within men's symptom network: Depending on the type of endorsed body ideals and perceived discrepancies in Community 4 (for example, as part of “bulking” and “cutting” phases during intense training), symptom activation could shift from ED‐specific forms of fear and avoidance (Community 2) and shape/weight overvaluation (Community 3) towards the “male” body shaping and rule‐based dieting (Community 1) or selective eating (Community 5) behaviours. Thus, men's EDs may best be characterised within a dynamic continuum ranging from muscularity‐oriented and otherwise selective (ARFID‐related) symptoms to more “traditional” ED symptoms (Schaefer et al., 2021). However, these assumptions require further empirical validation.

Finally, we found no indication that symptom network structures differed based on sample characteristics. Although men with an increased ED risk were characterised by increased ED symptoms (including orthorexic behaviours), leanness discrepancies, and muscle dysmorphic symptoms, there was no indication that ED risk affected the global network structure or strength. However, the test had limited power due to the size of the low‐risk group, and we were unable to evaluate the stability of centrality estimates. Thus, whether low‐ and high‐risk groups only differ in the severity of disordered eating symptoms requires further investigation.

### 4.1 Limitations

Our findings are subject to limitations. Although we could largely corroborate previously obtained network structures in men, we did not specify a specific network and its required sample size a priori. Replicating a similar network (e.g., with 30 nodes and 40% non‐zero edges) with a power 0.75, under the assumption that symptoms are connected randomly, would require a more than tenfold increase in sample size (4,239), whereas limiting the network to the three most central symptoms per community (15 nodes) would reduce that estimate to 1002 (Constantin et al., 2023). Considerably fewer participants are required when assuming a non‐random network architecture (Constantin & Cramer, 2019), consistent with the idea of symptoms “clustering” into specific communities. Still, the current network configuration should be interpreted cautiously, as replications using the same underlying symptoms are needed.

Moreover, although we specifically aimed to include a broader range of disordered eating symptoms (and largely reproduced previously found symptom networks in men), our findings are equally limited by the set of included nodes. For example, we did not include an assessment of steroid or other types of substance abuse (Prnjak et al., 2021) and thus may have missed further symptom associations.

It is further important to acknowledge the cross‐sectional nature of our study. Thus, while centrality estimates (and bridge centrality specifically) could be informative in identifying diagnostic and screening targets, we cannot ascertain the causal direction of influence between the symptoms nor draw conclusions about which specific factors could facilitate the development of an ED in men. Further longitudinal investigations are required.

Finally, our and other network analyses among men that included muscularity concerns thus far only considered non‐clinical samples. While we assessed ED risk using a screening instrument, we did not include a structured diagnostic interview. Thus, it remains an open question whether muscularity concerns play a similar central role among men with clinically diagnosed EDs or in those in treatment. Specifically, treatment could either ameliorate or, temporarily, aggravate body image concerns and restrictive or selective eating tendencies (e.g., due to prescribed weighing or structured meal schedules). We must also note that a majority of participants were classified as at risk, which raises questions about the validity of the classification. We initially speculated that the convenience sample could have been motivated to participate due to the subject of the study itself, leading to a selection bias. However, the Prolific sample, which offered a monetary incentive, yielded similarly high rates of at‐risk men, suggesting that the screening instrument might have been overly sensitive. Given that other factors, so‐called “external field” factors (Monteleone & Cascino, 2021), such as sociodemographic features, may also affect ED presentation (Halbeisen, Brandt, & Paslakis, 2022; Traut et al., 2023), further investigations on network reproducibility among clinical and more diverse samples are required.

### 4.2 Conclusion

The limitations notwithstanding, our findings provide several important conclusions: Muscularity concerns emerged as central in men's disordered eating networks, but muscularity‐related behaviours may be best considered a part of a broader cluster of body shaping and dieting behaviours that may be more frequently observed among men. Moreover, symptom bridges emerged along affective dimensions. Given that behaviours and ideals are subject to sociocultural norms and changes, the burden associated with any of these behaviours might prove particularly informative for the detection of potential ED cases, including among men.

## PUBLIC SIGNIFICANCE STATEMENT

Eating disorders increasingly affect men, but their symptoms may go unnoticed due to stereotypical perceptions and gender differences in symptom presentation. This study used network analysis to corroborate the importance of muscularity concerns in men's symptom networks, and suggests that behaviours directed towards increasing muscularity are part of a broader cluster of male body shaping and strict dieting behaviours. These findings can help to improve the detection of eating disorders in men.

## CONFLICT OF INTEREST STATEMENT

All authors confirm there being no conflicts of interest.

## Supporting information

Figure S1

## Data Availability

The data that support the findings of this study are available from the corresponding author upon reasonable request.
