# Supplementary material for: Investigating the structure of disordered eating symptoms in adult men: A network analysis
Source: Eur Eat Disord Rev. 2024 Aug 12;33(1):80–94. doi: 10.1002/erv.3131 (PMC11617807; doi:10.1002/erv.3131)
Supplement: Supplementary file 1 — Figure S1 [file ERV-33-80-s001.docx]

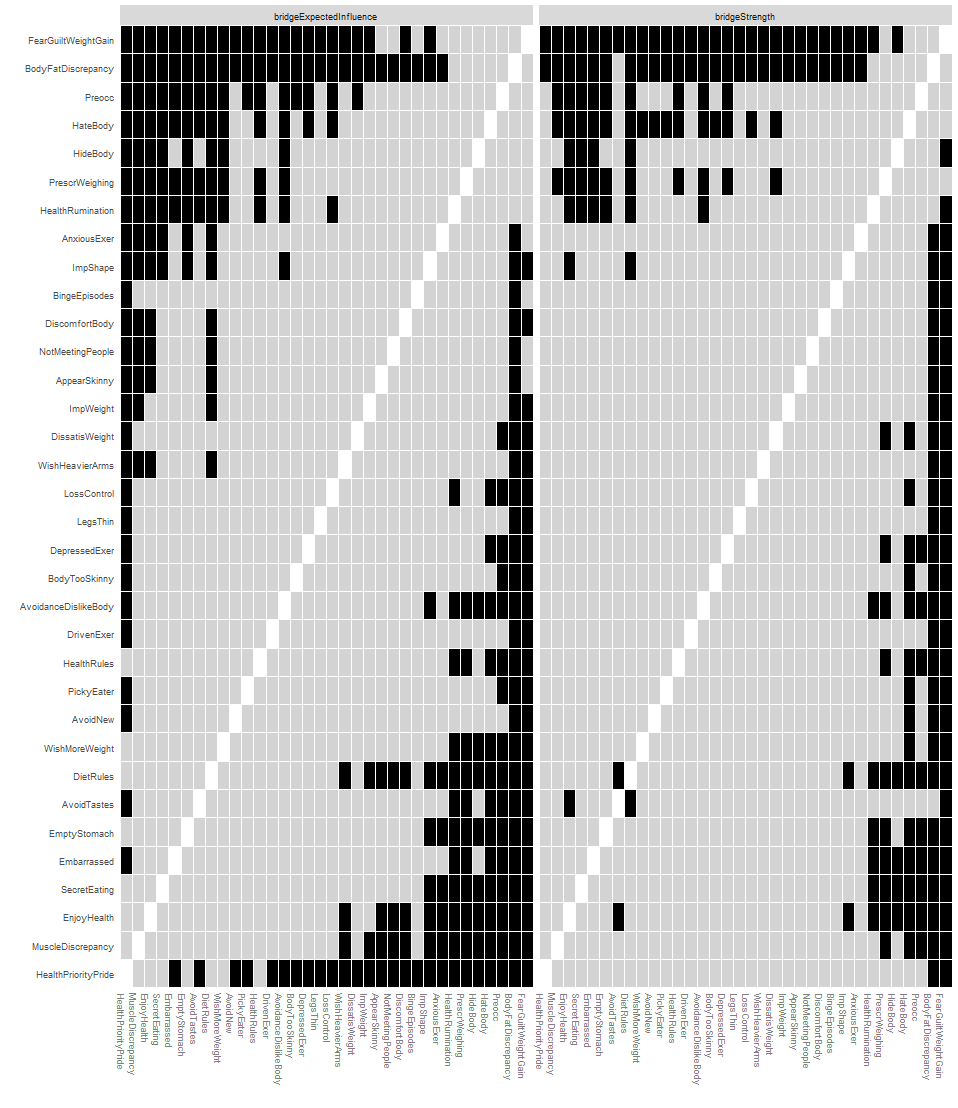


***Figure S1***. Results of bridge centrality difference tests. Black boxes indicate significant differences between edges in terms of centrality (expected influence and strength, respectively); gray boxes indicate nonsignificant differences.
